# CHOLECYSTECTOMY WITH INTRAOPERATIVE ENDOSCOPIC RETROGRADE CHOLANGIOPANCREATOGRAPHY: DOES THE ORDER MATTER?

**DOI:** 10.1590/0102-6720202400023e1816

**Published:** 2024-08-19

**Authors:** João de Bona Castelan, Arthur Pizzolatti Zapelini, Felipe Antônio Cacciatori, Bruno Zilberstein

**Affiliations:** 1 Hospital São José, General Surgery Service – Criciúma (SC), Brazil; 2 Santa Casa de Misericórdia, Hepatobiliopancreatic and Liver Transplant Service – Porto Alegre (RS), Brazil; 3 Universidade Federal do Rio Grande do Sul, Master in Surgical Sciences – Porto Alegre (RS), Brazil; 4 Universidade de São Paulo, Faculty of Medicine, Cancer Institute – São Paulo (SP), Brazil.

**Keywords:** Biliary Tract Diseases, Biliary Tract Surgical Procedures, Cholangiopancreatography, Endoscopic Retrograde, Cholecystectomy, Laparoscopic, Doenças Biliares, Procedimentos Cirúrgicos do Sistema Biliar, Colangiopancreatografia Retrógrada Endoscópica, Colecistectomia Laparoscópica

## Abstract

**BACKGROUND:**

The recommended treatment for cholecystocholedocholithiasis is cholecystectomy (CCT) associated with endoscopic retrograde cholangiopancreatography (ERCP). CCT with intraoperative ERCP is associated with higher success rates and lower hospital stays and hospital costs. However, some case series do not describe the exact methodology used: whether ERCP or CCT was performed first.

**AIMS:**

Verify if there is a difference, in terms of outcomes and complications, when intraoperative ERCP is performed immediately before or after CCT.

**METHODS:**

This is a retrospective case-control study analyzing all patients who underwent CCT with intraoperative ERCP between January 2021 and June 2022, in a tertiary hospital in southern Brazil, for the treatment of cholecystocholedocholithiasis.

**RESULTS:**

Out of 37 patients analyzed, 16 (43.2%) underwent ERCP first, immediately followed by CCT. The overall success rate for the cannulation of the bile duct was 91.9%, and bile duct clearance was achieved in 75.7% of cases. The post-ERCP pancreatitis rate was 10.8%. When comparing the "ERCP First" and "CCT First" groups, there was no difference in technical difficulty for performing CCT. The "CCT First" group had a higher rate of success in bile duct cannulation (p=0.020, p<0.05). Younger ages, presence of stones in the distal common bile duct and shorter duration of the procedure were factors statistically associated with the success of the bile duct clearance. Lymphopenia and cholecystitis as an initial presentation, in turn, were associated with failure to clear the bile duct.

**CONCLUSIONS:**

There was no significant difference in terms of complications and success in clearing the bile ducts among patients undergoing CCT and ERCP in the same surgical/anesthetic procedure, regardless of which procedure was performed first. Lymphopenia and cholecystitis have been associated with failure to clear the bile duct.

## RESUMO

**RACIONAL:**

O tratamento recomendado para colecistocoledocolitíase é a colecistectomia (CCT) associada à colangiopancreatografia endoscópica retrógrada (CPRE). A CCT com CPRE intraoperatória está associada a maiores taxas de sucesso e menor tempo de permanência hospitalar e menos custos hospitalares. No entanto, algumas séries de casos não descrevem a metodologia exata utilizada: se a CPRE ou a CCT foi realizada primeiro.

**OBJETIVOS:**

Verificar se há ou não diferença, em termos de resultados e complicações, quando a CPRE intraoperatória é realizada imediatamente antes ou após a CCT.

**MÉTODOS:**

Estudo caso-controle, retrospectivo, que analisou todos os pacientes submetidos à CCT com CPRE intraoperatória, entre janeiro de 2021 e junho de 2022, em um hospital terciário do Sul do Brasil, para tratamento de colecistocoledocolitíase.

**RESULTADOS:**

Dos 37 pacientes analisados, 16 (43,2%) foram submetidos primeiro à CPRE, seguida imediatamente pela CCT. A taxa global de sucesso para a canulação do ducto biliar foi de 91,9% e a desobstrução do ducto biliar foi alcançada em 75,7% dos casos. A taxa de pancreatite pós-CPRE foi de 10,8%. Ao comparar os grupos "CPRE Primeiro" e "CCT" primeiro", não houve diferença na dificuldade técnica para realização da CCT. O grupo "CCT primeiro" teve maior taxa de sucesso na canulação do ducto biliar (p=0,020, p<0,05). Idades mais jovens, presença de cálculos na via biliar comum distal e menor duração do procedimento foram fatores estatisticamente associados ao sucesso na desobstrução da via biliar. A linfopenia e a colecistite como apresentação inicial, por sua vez, foram associadas à falha na desobstrução do ducto biliar.

**CONCLUSÕES:**

Não houve diferença significativa em termos de complicações e sucesso na desobstrução das vias biliares entre pacientes submetidos a CCT e CPRE no mesmo procedimento cirúrgico/anestésico, independentemente de qual procedimento foi realizado primeiro. Linfopenia e colecistite são associadas à falha na desobstrução do ducto biliar.

## INTRODUCTION

The treatment of cholelithiasis with choledocholithiasis is complex and can be performed by cholecystectomy (CCT) with choledochotomy^27^, either laparoscopically or open, or by CCT combined with endoscopic approach of the bile duct, using endoscopic retrograde cholangiopancreatography (ERCP)^15,25,29^. However, the ideal time to perform CCT, before^16^, during^14,18^ or after^30,31^ ERCP, is a controversial topic and is still under discussion in the literature^2,15,17,20,28,29,40^.

Performing intraoperative ERCP with CCT for timely treatment of cholelithiasis with choledocholithiasis is associated with higher rates of success, shorter hospital stays and lower hospital costs^8,18,20,21,28,33,40^. This indication, however, is limited to services in which the surgeon is qualified to perform both procedures or in which there is availability of an endoscopist to perform the procedure at the surgical center together with CCT^1,22^.

Some published case series on the topic mention the CCT being performed first, under general anesthesia, followed immediately by ERCP^14,18^. Others performed ERCP first, followed by CCT^21,28^. There are also series that use the laparoscopic rendezvous technique for intraoperative ERCP^10,26,32^. However, some of the series found do not describe the exact methodology^12,20,23,33^.

The objective of the present study is to verify whether or not there are differences in terms of outcomes and complications when ERCP is performed immediately before or after CCT, but in the same surgical/anesthetic procedure, in order to clarify this gap in the literature.

## METHODS

Retrospective case-control study, analyzing all patients who underwent CCT with ERCP in the same surgical/anesthetic procedure, between January 2021 and June 2022, in a tertiary general hospital in southern Brazil, in a total of 37 cases. The exclusion criteria, which were incomplete medical records and age under 18, did not discard any records. All patients had a preoperative diagnosis confirmed by magnetic cholangioresonance imaging of choledocholithiasis with cholelithiasis.

The hospital serves a population of around one million inhabitants, being a reference for patients with choledocholithiasis and receiving patients referred from other institutions or treated urgently. The hypothesis of choledocholithiasis is made during medical history and physical examination and confirmed by initial complementary tests, such as serum levels of bilirubin, amylase and canalicular enzymes, in addition to ultrasound^7,41^. Given this clinical picture, a specific investigation of the bile ducts is indicated, using magnetic cholangioresonance^41^.

Once the diagnosis of choledocholithiasis with cholelithiasis has been confirmed, the definitive treatment is determined through ERCP with CCT in the same surgical/anesthetic act, both procedures being performed by the same surgeon. In the operating room, after general anesthesia, the patient is positioned in the left lateral decubitus position and the first ERCP is performed and then repositioned to supine position to perform videolaparoscopic CCT surgery, or vice versa, with no clear reason to indicate one procedure or the other to be performed first. Prophylactic measures for post-ERCP pancreatitis are not used in the institution, such as vigorous hydration^5,38^, rectal indomethacin^6,33,35,36^, or any other methods^11^. Serum amylase is routinely dosed 6 hours later, to identify complications associated with the procedure.

The electronic medical records were retrospectively analyzed and the data tabulated comparing the variables and outcomes of patients allocated in the "ERCP first" or "CCT first" groups. Afterwards, patients were reallocated into the "Success in bile duct clearance" or "Without success in bile duct clearance" groups, in order to detail the findings of the casuistry. Data tabulation took place in Microsoft Excel software and statistical analysis was performed using IBM Statistical Package for the Social Sciences (SPSS) Statistics, version 18.0 of the software, through the construction of frequency distributions and comparisons between the dependent and independent variables. As measures of central tendency the measurements of averages and standard deviation were used, as well as the median and interquartile range. The Kolmogorov-Smirnov test was used to determine the normality or non-normality of comparative data, and Student's *t* or Mann-Whitney's U tests were used for the other analysis. Pearson's chi-square and likelihood ratio, with complementary evaluation of the analysis of residue and Cramer's V test were used. The definition employed for successful bile duct cannulation was the effective passage of the guidewire through the duodenal papilla with radioscopic confirmation of the bile duct catheterization.

The definition used for successfully clearing the bile duct was the absence of radioscopic images that could suggest the continuance of stones after appropriate procedures. The duration of the procedure was recorded from the time of beginning anesthetic induction, therefore including the airway management time for the anesthesiologist. The end of the procedure was the patient's extubation, which was successful in all cases analyzed. The definition used for post-ERCP pancreatitis is the occurrence of new epigastric pain associated with an increase in pancreatic enzymes three times higher than the regular upper limit, within 24 hours of the procedure, and requiring hospitalization for more than two nights^11,39,40^. When the occurrence of isolated hyperamylasemia was identified without clinical alterations or need to remain hospitalized, the condition was defined as asymptomatic hyperamylasemia^9^.

The study was approved by the institution's Ethics Committee, duly registered on Plataforma Brasil under Certificate of Presentation for Ethical Appreciation (CAAE) 59955722.3.0000.5364, report number 5,525,543. The Strengthening the Reporting of Observational Studies in Epidemiology (STROBE) checklist for case control studies was carefully observed^13^.

## RESULTS

Of the 37 patients analyzed, 16 (43.2%) underwent ERCP followed by CCT and 21 (56.8%) underwent CCT followed by ERCP, both procedures in the same anesthetic/surgical procedure. Table 1 demonstrates the characteristics of the sample. The overall success rate in bile duct clearance was 75.7% and the rate of complications attributed to ERCP was 10.8%, corresponding to the four cases of post-ERCP pancreatitis. The Sugrue score was used to classify the observed difficulty during CCT and no case of extreme difficulty was identified (grade D), so that the majority of cases (48.6%) were considered easy to perform.

**Table 1:** Sample characteristics.

Characteristics		n=37
Age (years)*		46.5±23.5
Gender†		
	Male	11 (29.7)
	Female	26 (70.3)
BMI*		27.8±4.7
Axillary temperature*		36.2±0.4
Hematocrit*		39.2±7.3
Amylase at arrival‡		68.0 (53.5–130.0)
Leukocytes at arrival*		9588.6±4476.5
Lymphocytes at arrival*		1740.3±1066.3
Total bilirubin at arrival‡		4.6 (1.25–9.95)
Number of stones at the MR‡		2 (1.0–4.0)
Size of the largest stone (millimeters)*		5.9±3.5
Stones in the distal common bile duct?†		
	Yes	15 (40.5)
	No	22 (59.5)
Cholecystitis as initial presentation?†		
	Yes	5 (13.5)
	No	32 (86.5)
Acute pancreatitis as initial presentation?†		
	Yes	5 (13.5)
	No	32 (86.5)
Interval between admission and procedure (days)*		6.6±4.9
Order of procedures†		
	ERCP followed by cholecystectomy	16 (43.2)
	Cholecystectomy followed by ERCP	21 (56.8)
Success in the cannulation of the bile duct?†		
	Yes	34 (91.9)
	No	3 (8.1)
Success in clearing the bile duct?†		
	Yes	28 (75.7)
	No	9 (24.3)
Papillotomy?†		
	Yes	35 (94.6)
	No	2 (5.4)
Total duration of the procedure (minutes)*		178.9±66.1
Number of stones removed‡		1 (0.0–2.0)
Sugrue†		
	A) Easy	18 (48.6)
	B) Moderate	14 (37.8)
	C) Hard	5 (13.5)
Post-ERCP amylase‡		94.0 (58.0–306.0)
Days admitted after procedure‡		1.0 (1.0–2.0)
General complications after procedure†		
	Post-ERCP pancreatitis	4 (10.8)
	Asymptomatic hyperamylasemia	3 (8.1)
	Coleperioneum	1 (2.7)
Complications greater than Clavien-Dindo II		1 (2.7)

* Values showed as average and standard deviation;

† Values showed as frequency and percentage;

‡ Values showed as median and interquartile amplitude. BMI: body mass index; MR: magnetic resonance; ERCP: endoscopic retrograde cholangiopancreatography.

There was a complication in only one case, which required treatment, which characterizes grade II in the Clavien-Dindo classification^7^. The patient underwent ERCP first and subsequently required laparoscopic reapproach on the second postoperative day due to choleperitoneum of 530 mL, secondary to leakage in the cystic duct clipping. This patient was discharged on the eighth postoperative day after the initial procedure, without other complications.

Table 2 demonstrates that, although there was no randomization or even targeted allocation of patients between the "ERCP First" or "Cholecystectomy First" groups, most variables did not differ significantly between groups, demonstrating a certain homogeneity between them. There was a significant difference in the variables "amylase on arrival", which was higher in the group undergoing CCT first (p=0.008, p<0.05), and in the variable "bile duct cannulation success", which was significantly more successful in the "CCT first" group (p=0.020, p<0.05), this relationship being statistically confirmed by residue analysis and Cramer's V test (p=0.038, p<0.05).

**Table 2:** Comparison between endoscopic retrograde cholangiopancreatography first and cholecystectomy first groups.

Variables		ERCP firstn=16	CCT firstn=21	p-value	
Age (years)*		38.0±18.1	45.0±27.0	0.914†	
Gender‡					
	Male	3 (18.8)	8 (38.1)	0.195§	
	Female	13 (81.3)	13 (61.9)		
BMI*		28.5±5.0	28.3±5.1	0.945†	
Amylase at arrival//		55.0 (48.2–66.7)	92 (68.7–273.0)	0.008†	
Leukocytes at arrival*		9,666.7±4124.7	8739.6±3228.1	0.091†	
Lymphocytes at arrival*		1,970.1±1487.9	1548.6±834.2	0.629†	
Total bilirubin at arrival//		6.1 (1.9–9.5)	3.5 (1.2–4.6)	0.027†	
Number of stones in the common bile duct//		1.0 (1.0–6.0)	2.0 (1.0–4.0)	0.534†	
Success in cannulation?‡					
	Yes	13 (81.3)	21 (100.0)^a^	0.020§	
	No	3 (18.8)¶	0 (0.0)		
Success in clearing the bile duct?‡					
	Yes	12 (75.0)	16 (76.2)	0.933§	
	No	4 (25.0)	5 (23.8)		
Post-ERCP pancreatitis?‡					
	Yes	1 (6.3)	3 (14.3)	0.423§	
	No	15 (93.8)	18 (85.7)		
Sugrue et al.37‡					
	A	6 (37.5)	12 (57.1)	0.458§	
	B	7 (43.8)	7 (33.3)		
	C	3 (18.8)	2 (9.5)		

* Values showed as average and standard deviation;

† Value obtained by Mann-Whitney's U Test;

‡ Values showed as frequency and percentage;

§ Value obtained by the χ^2^ likelihood-ratio test;

// Values showed as median and interquartile amplitude;

¶ Values with statistical significance after the analysis of residue and significance p=0,038 in Cramer's V test.

ERCP: endoscopic retrograde cholangiopancreatography; CCT: cholecystectomy. BMI: body mass index.

Regarding the success in clearance of the bile duct, Table 3 demonstrates that younger ages, presence of stones in the distal common bile duct and shorter time duration of the procedure were factors statistically associated with successful bile duct clearance. Lymphopenia and cholecystitis as an initial presentation, in turn, were associated with failure in the bile duct clearance. There was no significant difference in the other variables studied.

**Table 3:** Comparison between groups with and without success in clearing the bile duct.

Variables		Success in clearingn=28	Without success in clearingn=9	p-value
Age (years)*		35.3±19.4	61.8±24.6	0.073†
Gender‡					
	Male	9 (32.1)	2 (22.2)	0.563§
	Female	19 (67.9)	7 (77.8)	
BMI*		28.5±5.3	28.0±4.2	0.665//
Amylase when admitted¶		77 (58.5–410.0)	66 (42.0–92.0)	0.101†
Leukocytes when admitted*		8066.9 ± 2817.2	12347.1 ± 3967.2	0.137//
Lymphocytes when admitted*		2027.2±1162.8	835.4±501.9	0.004†
Total bilirubin at arrival¶		4.3 (1.4–6.1)	4.6 (1.2–7.6)	0.599†
Number of stones in the common bile duct¶		1 (1.0–3.5)	2 (1.0–10.0)	0.392†
Size of the larges stones in mm*		5.2±3.2	6.2±3.7	0.410†
Duration of Procedure in min*		166.0±60.8	241.0±78.9	0.011†
Post-ERCP Amylase¶		78.0 (58.0–156.5)	402.0 (80.0–438.0)	0.396†
Stones in the distal common bile duct?‡					
	Yes	14 (50.0)#	1 (11.1)	0.027§
	No	14 (50.0)	8 (88.9)#	
Cholecistitis as initial presentation?‡					
	Yes	1 (3.6)	4 (44.4)**	0.004§
	No	27 (96.4)**	5 (55.6)	
Pancreatitis as initial presentation?‡					
	Yes	5 (17.9)	0 (0.0)	0.082§
	No	23 (82.1)	9 (100)	
Post-ERCP pancreatitis?‡					
	Yes	4 (14.3)	0 (0.0)	0.123§
	No	24 (85.7)	9 (100.0)	

* Values showed as average and standard deviation;

† Value obtained by applying Mann-Whitney's U Test;

‡ Values showed as frequency and percentage;

§ Value obtained by applying the χ^2^ Likelihood-ratio Test;

// Value obtained by applying the t-student test;

¶ Values showed as median and interquartile amplitude;

# Values with statistical significance after the analysis of residue and significance p=0,038 in the Cramer V Test;

** Values with statistical significance after the analysis of residue and significance p=0,002 in the Cramer V Test. BMI: body mass index;

ERCP: endoscopic retrograde cholangiopancreatography.

## DISCUSSION

The study presents a series of cases of cholelithiasis associated with choledocholithiasis, treated with ERCP and CCT in a single act. The overall rate of success in bile duct cannulation was 91.9%, bile duct clearance of 75.7%, post-ERCP pancreatitis of 10.8%, all data remaining within the scope defined in global literature^1,3-5,9,13,19,20,24,34^. This data corroborates the safety and effectiveness of the procedures, already suggested by other previously published articles^12-14,18,20,33,39^.

The evaluation of success rates and complications when comparing patients who underwent ERCP first or CCT first, in the same surgical/anesthetic act, demonstrated that there was a significant association between performing cholecystectomy first and successful cannulation of the bile duct. There was no difference between the groups when evaluating difficulty in performance of cholecystectomy, the rate of post-ERCP pancreatitis and success in bile duct clearance^39^.

These findings oppose the empiricism that performing ERCP first would make subsequent CCT difficult due to gaseous distension of the digestive tract, as well as that CCT first could be associated with biliary fistulas due to increased pressure caused by ERCP in the newly clipped cystic duct.

The technical steps to be observed in carrying out safe CCT, widely disseminated by Strasberg et al.^36^, culminated in the development of CCT intraoperative difficulty grading systems such as Sugrue et al.^37^. Although not all patients presented acute cholecystitis, the authors considered this score adequate to assess the technical difficulty in CCT and check its association with ERCP first.

Unlike other cases, the technique of laparoendoscopic rendezvous was not used in this series. This technique consists of performing CCT first with laparoscopic passage of a guidewire through the cystic duct towards the duodenum, running through the common bile duct and protruding through the major papilla. At this time, ERCP is performed by endoscopically identifying a guidewire and guided cannulation of the bile duct. This technique virtually eliminates the failure of bile duct catheterization, as well as the occurrence of post-ERCP pancreatitis^12,26,32,33^.

By demonstrating similar outcomes despite the order adopted, this study may serve to recommend performing ERCP first, because if failure of catheterization of the bile duct occurs using this, then CCT with retrograde catheterization of the cystic duct can be performed through laparoendoscopic rendezvous technique, allowing ERCP to be performed. If there is still a failure in the catheterization or bile duct clearance, there remains the option to laparoscopically explore the main bile duct. In cases of intraoperative diagnosis of choledocholithiasis using transcystic cholangiography during CCT, the option of laparoendoscopic rendezvous technique could also be used, increasing the success rate of ERCP. These statements, however, require confirmation by new, prospective and randomized studies.

## CONCLUSIONS

This research did not reveal a significant difference in terms of complications and success in bile duct clearance among patients undergoing CCT and ERCP in the same surgical/anesthetic procedure, regardless of which procedure was performed first. A higher success rate was registered in bile duct cannulation in patients undergoing CCT first. Lymphopenia and cholecystitis were associated with failure in bile duct clearance.
